# The time-course augmentation of perivascular space enlargement in the basal ganglia among a community-dwelling elder population

**DOI:** 10.1007/s11604-024-01595-3

**Published:** 2024-06-19

**Authors:** Yasuhiro Sugai, Toshitada Hiraka, Akiko Shibata, Ayato Taketa, Taiyo Tanae, Yosuke Moriya, Masanori Komatsu, Chifumi Iseki, Yasuyuki Ohta, Masafumi Kanoto

**Affiliations:** 1 Division of Diagnostic Radiology, Department of Radiology, Faculty of Medicine, Yamagata University, 2-2-2 Iida-Nishi, Yamagata, 990-9585 Japan; 2 Department of Behavioral Neurology and Cognitive Neuroscience, Tohoku University Graduate School of Medicine, Sendai, Japan; 3 Division of Neurology and Clinical Neuroscience, Department of Internal Medicine III, Faculty of Medicine, Yamagata University, Yamagata, Japan

**Keywords:** Cerebral small vessel disease, Glymphatic system, Magnetic resonance imaging, Perivascular space

## Abstract

**Purpose:**

We examined whether time-course augmentation of perivascular space enlargement in the basal ganglia (BG-PVS) reflected cerebral small vessel disease (CSVD) severity by considering white matter hyperintensity lesion (WMHL) as an indicator for CSVD.

**Materials and methods:**

This study population included 416 older participants from a community-based cohort. They participated in magnetic resonance imaging (MRI) studies more than once during the study period. The grades for BG-PVS and WMHL were evaluated by visual rating scales; BG-PVS time-course augmentation in 4–9 years was also evaluated. At baseline, the participants were asked about their smoking and drinking history, and medical history. They also underwent a blood examination and their office blood pressure (BP) examination. In addition, 24-h ambulatory BP monitoring was also performed within the study period.

**Results:**

Of the 416 participants, 48 participants (11.5%) had BG-PVS time-course augmentation. The participants with BG-PVS augmentation had significantly lower LDL levels, hyper-nighttime BP, and lower nighttime BP fall in univariate analysis (*p* = 0.03, *p* = 0.03, *p* = 0.003, respectively). In multivariate analysis, lower nighttime BP fall and male sex showed significance (*p* = 0.02, 0.03, respectively). Additionally, BG-PVS time-course augmentation was significantly associated with subsequent WMHL severity in univariate analysis (*p* < 0.001), which remained significant in multivariate analysis adjusted by imaging and demographic factors (*p* = 0.03). In multivariate analysis, additionally adjusted by the clinical factors, the significance disappeared (*p* = 0.07).

**Conclusion:**

This study revealed that the lower nighttime BP fall in ambulatory blood pressure monitoring was a factor significantly associated with BG-PVS augmentation. Moreover, the BG-PVS time-course augmentation would be a notable finding that was associated with the subsequent WMHL.

## Introduction

Perivascular space (PVS) is a small fluid-filled space around the penetrating vessels. It becomes visible on brain magnetic resonance imaging (MRI) if it enlarges. Among healthy populations, PVS becomes more visible with age [1, 2]. PVS is thought to be one of the elements composing the glymphatic system, which is a material and fluid transport mechanism through the cerebrospinal fluid around the brain and interstitial fluid in the brain parenchyma [3, 4]. This system plays an important role in maintaining optimal situations in the intracranial environment [4]. The enlargement of the PVS (EPVS) implies the impairment of the glymphatic system, which was revealed by using the noninvasive method [5].

Cerebral small vessel disease (CSVD) is a disease that is suspected to be associated with the impairment of the glymphatic system [6]. CSVD is a disorder caused by intra-axial small vessels, including arteriole, capillary, and venule [7]. The prevalence rate of CSVD increases depending on age, and CSVD relates to cerebral infarction, intracranial hemorrhage, and dementia among the older population [7]. In addition to white matter hyperintensity lesions (WMHL), lacunes and micro-bleeding, EPVS is an MRI finding suggesting CSVD [8]. Previous studies have revealed that EPVS had a significant association with hypertension [2, 9–12], which is one of the risk factors for CSVD [13, 14]. Together with the fact that both EPVS and CSVD become frequent with age, EPVS, which is more frequently identified among the elderly population, would reflect the worsening trend for CSVD from the impairment of small vessels by hypertension.

In previous studies, it was revealed that EPVS at a single point in time is related to various clinical conditions and diseases, including hypertension [2, 9–12], lacunar infarction [12, 15, 16], and vascular dementia [17, 18]. However, it remains unclear whether the time-course augmentation of EPVS has any clinical significance. Some studies have revealed that EPVS only in basal ganglia, not in white matter, was associated with hypertension [2, 10], higher daytime blood pressure (BP) [9], and lacunar infarction [16], which implied that PVS in the basal ganglia (BG-PVS) would reflect hypertensive vasculopathy. In this study, we evaluated the following two points to search whether the time-course augmentation of BG-PVS using MRI has any significance. First, we examined which clinical factors were related to the BG-PVS time-course augmentation. Next, we examined whether BG-PVS time-course augmentation was associated with the severity of WMHL in the subsequent MRI study. That is, we examined whether BG-PVS augmentation reflected CSVD severity by considering WMHL as an indicator for CSVD as per a previous study [19].

## Materials and methods

This retrospective study was approved by our institution’s ethics committee; we obtained informed consent from all participants.

### Subjects

**Fig. 1:**
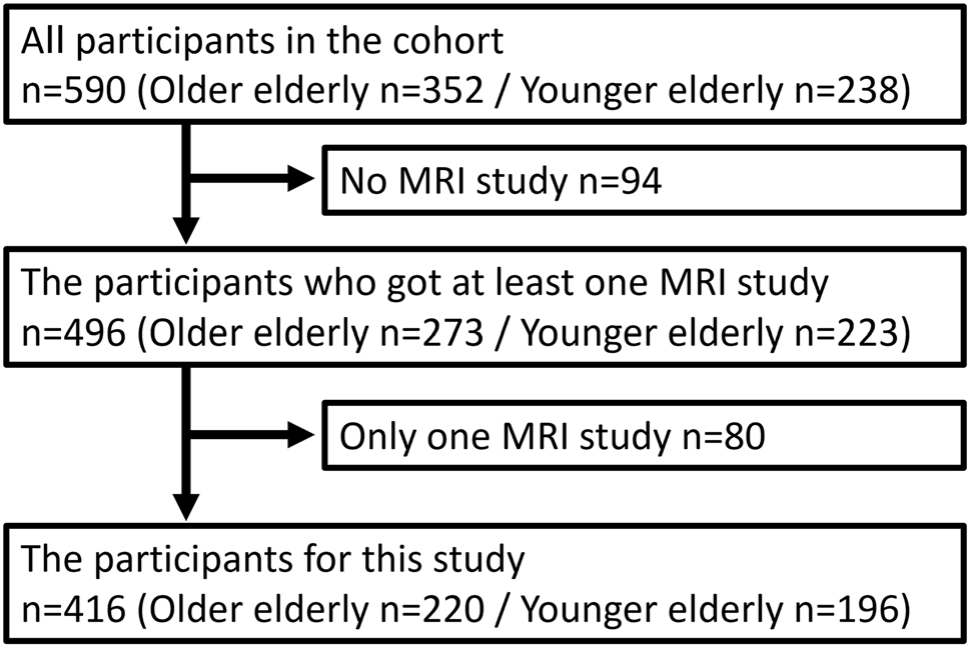
The protocol and number of participants. Among all participants in the cohort, 94 participants without MRI study and 80 participants with one MRI study were excluded. The remaining 416 participants were targeted in this study

The study population was extracted from the previously described Takahata cohort study [20]. In short, this is a prospective community-based cohort study conducted in 2000. The whole cohort consisted of two birth cohorts, with participants born between April 1930 and March 1931 or between April 1941 and March 1942. In this study, the former was named the older elderly, and the latter was named the younger elderly. The participants were called for periodic medical examinations, including MRI studies, unless they had any contraindications. In this study, we evaluated MRI studies performed in 2000, 2004, and 2008 for the older elderly, and these were performed in 2002, 2007, and 2011 for the younger elderly. Because not all the participants had undergone all the MRI studies because of personal reasons despite our recruitment, the timing of the MRI studies for each participant varied. Table 1 shows how many participants belonged to each combination of the MRI studies. Of all the participants, 496 individuals had taken part in at least one MRI study. The 80 participants who had an MRI study at only one time were excluded because the time-course change in MRI findings could not be evaluated. The remaining 416 participants who had been involved in MRI studies two or three times were included in this study. The protocol and number of participants are summarized in Fig. 1.

**Table 1:** The number of participants who belong to each combination of MRI studies

Group	MRI study				Number of participants	
	First	Second	Third	All (*n* = 590)	Older elderly (*n* = 352)	Younger elderly (*n* = 238)
1	P	P	P	303	148	155
2	P	P	N	47	25	22
3	P	N	P	65	46	19
4	N	P	P	1	1	0
			Sum	416	220	196
5	P	N	N	75	49	26
6	N	P	N	4	4	0
7	N	N	P	1	0	1
8	N	N	N	94	79	15

*N* not performed, *P* performed

The 416 participants in Groups 1–4 were included in this study

### MRI acquisition

**Fig. 2:**
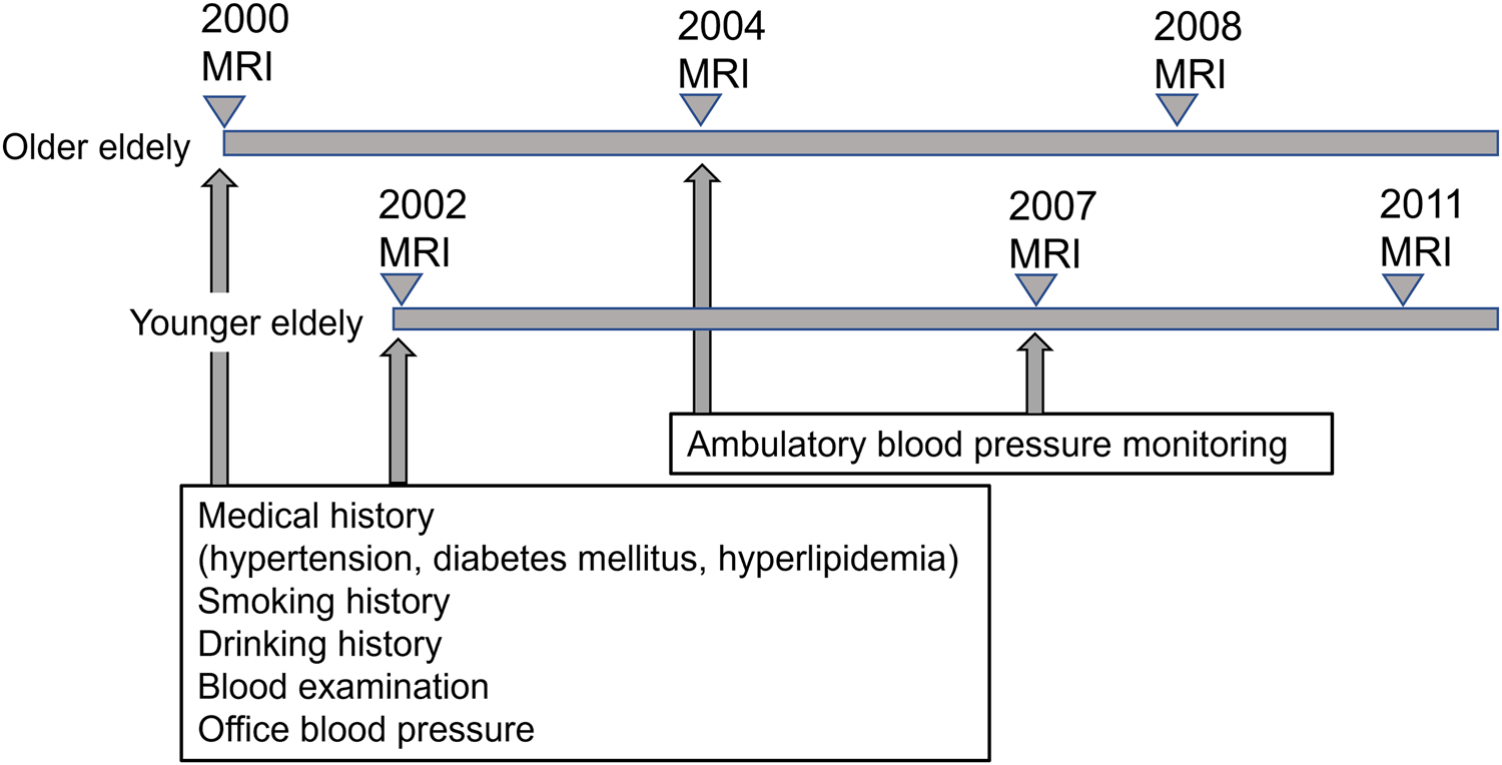
Overview of this study

All subjects were scanned on the same 0.3 T MRI system (AIRIS, Hitachi, Japan) in the same hospital. The protocol included 10 mm-thick axial sections of T1-weighted images (T1WI), T2-weighted images (T2WI), and fluid-attenuated inversion recovery (FLAIR) images, which were acquired using the following parameters: (1) T1WI: repetition time (TR)/echo time (TE) = 500/20 ms, flip angle (FA) = 90˚, matrix = 256 × 256, (2) T2WI: TR/TE = 3500/112 ms at the first study and TR/ TE = 4000/117 ms at the second and third studies, FA = 90˚, matrix = 256 × 256, (3) FLAIR: TR/TE/ inversion time (TI) = 6700/117/1200 ms at the first study and TR/TE/TI = 8000–8500/117/2000 ms at the second and third studies, matrix = 256 × 256. The participants included in this study underwent two or three MRI studies, so the time interval between the first and last study varied from 4 to 9 years (Table 1; Fig. 2).

### Perivascular space (PVS) assessment

One board-certified radiologist with 8 years of experience (Y.S.) assessed BG-PVS severity on each MRI study in all the participants as follows. PVS was defined as a round, oval, or linear lesion approximately < 3 mm with the same signals to the cerebrospinal fluid (CSF) [8]. The number of PVS was counted on T2WI. The FLAIR image was referred to exclude chronic ischemic lesions with hyperintensity. We divided BG-PVS severity into four grades based on a previously described visual rating scale: grade 1: < 5 PVS; grade 2: 5–10 PVS; grade 3: > 10 PVS but still countable; grade 4: too many PVS to count and cribriform on the slice containing the greatest number of PVS [21]. We also evaluated whether BG-PVS had time-course augmentation during the follow-up period. If the number of BG-PVS increased or BG-PVS became larger when visually compared with the former study, it was defined that BG-PVS time-course augmentation was present. To evaluate of the inter-rater reliability, another board-certified radiologist with 13 years of experience (T.H.) assessed BG-PVS severity on a subset of 40 participants, and a training radiologist with 2 years of experience (Y.M.) assessed whether BG-PVS time-course augmentation was observed during the follow-up period on another subset of 40 participants. The raters were blinded to all clinical factors on PVS assessment.

### White matter hyperintensity lesion (WMHL) assessment

The same radiologist (Y.S.) rated WMHL based on the Fazekas scale [22]. Periventricular lesions and deep white matter lesions were separately rated on the FLAIR images, as follows. Periventricular lesions were classified as grade 0 when absent, grade 1 when there was a cap or pencil-thin lining, grade 2 when there was a smooth halo, or grade 3 when irregular and extending into the deep white matter. Deep white matter lesions were classified as grade 0 when absent, grade 1 when punctate foci were present, grade 2 when beginning foci confluence, or grade 3 when present over a large confluent area. Similar to BG-PVS assessment, the other board-certified radiologist with 13 years of experience (T.H.) assessed WMHL severity on the subset of 40 participants to evaluate the inter-rater reliability. The raters were blinded to all clinical factors on WMHL assessment.

### Clinical evaluation

At the first examination, the participants were checked for their cardiovascular risk factors as follows. They were asked about their medical history, including hypertension, diabetes mellitus, and hyperlipidemia. They were also asked about their smoking and drinking histories. In addition, they had a blood examination for cardiovascular risk factors, in which plasma sugar, triglyceride, total cholesterol, and high-density lipoprotein cholesterol (HDL-C) were evaluated. Low-density lipoprotein cholesterol (LDL-C) was calculated using the Friedewald formula. The participants were checked for their office BP. Moreover, at the second examination, ambulatory blood pressure monitoring (ABPM) was performed using an automatic device. BP was recorded once every hour for 24 h. The nighttime BP fall was defined as [1 − (nighttime mean BP)/(daytime mean BP)] × 100 (%). Figure 2 shows the overview of this study.

### Statistical analysis

**Fig. 3:**
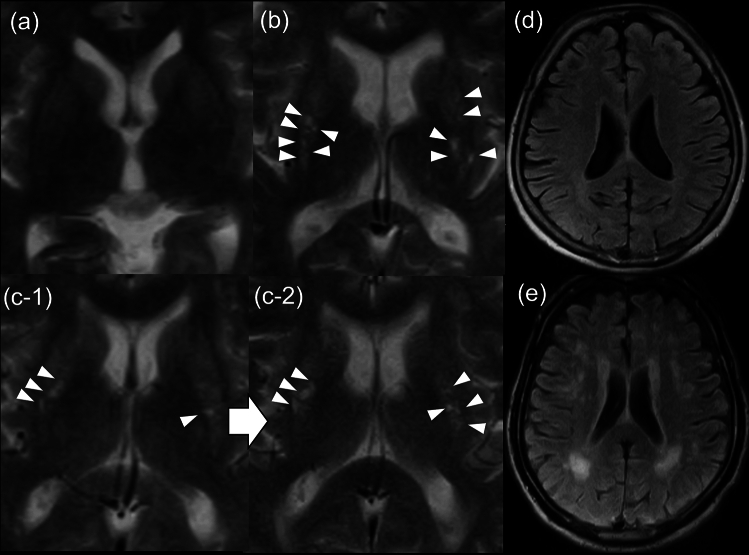
Representative examples of BG-PVS or WMHL severity and the time-course augmentation. Small arrowheads indicate enlarged PVSs and large arrow between images indicates time passage. **a**, **b** Low- and high-grade BG-PVS, respectively. **c-1**, **c-2** BG-PVS augmentation, that is, the BG-PVS counts increase in the study period. **d** Low-grade WMHL. **e** High-grade WMHL. *BG* basal ganglia, *PVS* perivascular space, *WMHL* white matter hyperintensity lesion

All categorical variables were expressed as frequency and percentage, and continuous variables were expressed as the mean and standard deviation. We dichotomized BG-PVS severity into low and high grades according to the four-point grading system. The former included grades 1 and 2, the latter includes grades 3 and 4 [10]. We also dichotomized WMHL severity into a low or high grade according to the Fazekas score. The latter included the participants with grade 3 periventricular lesions or grade 2 or more deep white matter lesions, and the former included the others [23]. Figure 3 shows representative examples of BG-PVS and WMHL with low and high grades, and BG-PVS time-course augmentation. First, inter-rater reliability was assessed using a Cohen’s kappa coefficient. The level of reliability was determined according to the criteria by Landis JR et al. [24]: < 0, poor; 0–0.20, slight; 0.21–0.40, fair; 0.41–0.60, moderate; 0.61–0.80, substantial; > 0.80, almost perfect. Second, the association between BG-PVS augmentation and clinical factors was examined using logistic regression analysis. Multivariate logistic regression analysis was performed with factors with a *p* value < 0.20 in univariate analysis in addition to sex and age. Finally, the association between the WMHL severity at the last MRI study and BG-PVS time-course augmentation was assessed using logistic regression analysis. In multivariate analysis 1, we adjusted for BG-PVS severity at the last MRI study in addition to sex- and age-defined groups because it had been reported that BG-PVS severity was related to WMHL at the same point in time [9–12]. For clinical factors, we took into consideration smoking history, hypertension, and diabetes mellitus, which had been reported as clinical factors relating to WMHL [25–27]. In multivariate analysis 2, we additionally adjusted for clinical factors, which were significant in univariate analysis. Because of the variety in the combination of participant MRI studies, accessory analyses were performed for the participants who each had two MRI studies. In the accessory analyses, the clinical factors that were significant in the univariate analysis among all participants were adjusted. Participants with missing data were excluded from each analysis.

A *p* value < 0.05 was considered statistically significant. All statistical analyses were performed by EZR (Jichi Medical University, Saitama, Japan) [28].

## Results

The inter-rater reliability for BG-PVS severity, WMHL severity and BG-PVS time-course augmentation was substantial (κ = 0.68, 95% CI 0.34–1.03), substantial (κ = 0.72, 95%CI 0.45–0.98), and moderate (κ = 0.59, 95%CI 0.26–0.93), respectively.

### Subject characteristics

This study population included 416 participants. The demographic, clinical, and radiographic characteristics are summarized in Table 2. Medical history, smoking, and drinking history, blood test, office BP, and 24-h ambulatory BP were available for 413 (99.3%), 413 (99.3%), 404 (97.1%), 398 (95.7%), and 329 (79.1%) participants, respectively.

**Table 2:** The characteristics of the 416 participants

	*N*		
Sex, male	416	*n* (%)	182 (43.8%)
Age, older elderly	416	*n* (%)	220 (52.9%)
Hypertension	413	*n* (%)	196 (47.5%)
Diabetes	413	*n* (%)	48 (11.6%)
Hyperlipidemia	413	*n* (%)	61 (14.8%)
Drinking history	413	*n* (%)	215 (52.1%)
Current smoker	413	*n* (%)	77 (18.6%)
Plasma sugar (mg/dl)	404	Mean (SD)	106.4 (24.5)
TG (mg/dl)	404	Mean (SD)	126.9 (82.8)
T-chol (mg/dl)	404	Mean (SD)	196.3 (34.7)
LDL-C (mg/dl)	404	Mean (SD)	115.3 (33.0)
HDL-C (mg/dl)	404	Mean (SD)	55.7 (16.2)
Office BP			
Systolic (mmHg)	398	Mean (SD)	139.5 (20.3)
Diastolic (mmHg)	398	Mean (SD)	77.7 (12.4)
24-h ambulatory BP (2nd examination)			
Daytime mean (mmHg)	329	Mean (SD)	104.3 (12.1)
Nighttime mean (mmHg)	329	Mean (SD)	92.5 (12.2)
Nighttime BP fall (mmHg)	329	Mean (SD)	11.0 (9.0)
MRI findings			
BG-PVS in the first study, high grade	415	*n* (%)	23 (5.5%)
BG-PVS in the second study, high grade	351	*n* (%)	24 (6.8%)
BG-PVS in the third study, high grade	369	*n* (%)	27 (7.9%)
WMHL in the last study, high grade	416	*n* (%)	87 (20.9%)

*BG-PVS* perivascular space in the basal ganglia, *BP* blood pressure, *HDL-C* high-density lipoprotein cholesterol, *LDL-C* low-density lipoprotein cholesterol, *T-chol* total cholesterol, *TG* triglyceride, *WMHL* white matter hyperintensity lesion

### Clinical factors associated with BG-PVS time-course augmentation

Of the 416 participants, 48 participants (11.5%) showed BG-PVS time-course augmentation. In univariate analysis, the participants with BG-PVS time-course augmentation had significant hyper-nighttime BP (OR 1.03, 95% CI 1.00–1.06), lower nighttime BP fall (OR 0.95, 95% CI 0.92–0.98), and lower LDL-C level (OR 0.99, 95% CI 0.98–1.00) when compared with participants without BG-PVS time-course augmentation (Table 3). The other factors did not have a significant association. In multivariate analysis, lower nighttime BP fall (OR 0.95, 95% CI 0.92–0.99) and male sex (OR 2.26, 95% CI 1.06–4.79) had significance (Table 3).

**Table 3:** The association between BG-PVS time-course augmentation and clinical factors

	*N*	BG-PVS augmentation					
		Negative	Positive	Univariate		Multivariate	
				Odds ratio	*p* value	Odds ratio	*p* value
Age (older elderly), *n* (%)	416	191 (51.9%)	29 (60.4%)	1.41 (0.77–2.61)	0.27	1.05 (0.48–2.26)	0.91
Sex (male), *n* (%)	416	155 (42.1%)	27 (56.2%)	1.77 (0.96–3.24)	0.07	2.26 (1.06–4.79)	0.03
Drinking history, *n* (%)	413	186 (51.0%)	29 (60.4%)	1.47 (0.80–2.71)	0.22		
Current smoker, *n* (%)	413	67 (18.4%)	10 (20.8%)	1.17 (0.56–2.47)	0.68		
Hypertension, *n* (%)	413	170 (46.6%)	26 (54.2%)	1.36 (0.74–2.48)	0.32		
Hyperglycemia, *n* (%)	413	58 (15.9%)	3 (6.2%)	0.35 (0.11–1.17)	0.09	0.43 (0.12–1.49)	0.18
Diabetes mellitus, *n* (%)	413	43 (11.8%)	5 (10.4%)	0.87 (0.33–2.32)	0.78		
Plasma sugar (mg/dl), mean (SD)	404	106.1 (24.3)	108.4 (26.3)	1.00 (0.99–1.01)	0.55		
TG (mg/dl), mean (SD)	404	127.3 (82.9)	123.9 (83.1)	1.00 (1.00–1.00)	0.79		
T-chol (mg/dl), mean (SD)	404	197.3 (35.0)	188.8 (31.8)	0.99 (0.98–1.00)	0.11	1.00 (0.98–1.03)	0.67
LDL-C (mg/dl), mean (SD)	404	116.6 (33.3)	105.6 (29.5)	0.99 (0.98–1.00)	0.03	0.99 (0.97–1.01)	0.38
HDL-C (mg/dl), mean (SD)	404	55.3 (15.9)	58.4 (18.1)	1.01 (0.99–1.03)	0.22		
Office BP							
Systolic (mmHg), mean (SD)	398	139.3 (20.3)	141.3 (20.0)	1.00 (0.99–1.02)	0.52		
Diastolic (mmHg), mean (SD)	398	77.64 (12.1)	77.8 (14.5)	1.00 (0.98–1.03)	0.92		
24-h ambulatory BP							
Daytime mean (mmHg), mean (SD)	329	104.3 (12.1)	104.4 (12.9)	1.00 (0.97–1.03)	0.98		
Nighttime mean (mmHg), mean (SD)	329	91.9 (12.3)	96.5 (10.1)	1.03 (1.00–1.06)	0.03	1.01 (0.98–1.05)	0.41
Nighttime BP fall (mmHg), mean (SD)	329	11.6 (8.7)	6.7 (10.8)	0.95 (0.92–0.98)	0.003	0.95 (0.92–0.99)	0.02

*BG-PVS* perivascular space in basal ganglia, *BP* blood pressure, *HDL-C* high-density lipoprotein cholesterol, *LDL-C* low-density lipoprotein cholesterol, *T-chol* total cholesterol, *TG* triglyceride

### Association between BG-PVS time-course augmentation and subsequent WMHL

In univariate analysis, BG-PVS time-course augmentation (OR 3.21, 95% CI 1.71–6.04), high BG-PVS grade at the last MRI study (OR 5.07, 95% CI 2.37–10.90), and age (OR 3.32, 95% CI 1.95–5.63) were significantly related to high WMHL grade at the last study. In multivariate analysis adjusted for the imaging and demographic factors, the significance remained in BG-PVS time-course augmentation (OR 2.22, 95% CI 1.07–4.59), high BG-PVS grade at the last MRI study (OR 3.31, 95% CI 1.40–7.83), and age (OR 3.10, 95% CI 1.80–5.35). In multivariate analysis additionally adjusted for the clinical factors, the significance disappeared in BG-PVS augmentation (OR 2.24, 95% CI 0.93–5.42) and high BG-PVS grade at the last MRI study (OR 2.61, 95% CI 0.88–7.70; Table 4). For the accessory analyses, BG-PVS time-course augmentation had significance in univariate and multivariate analysis adjusted for the imaging and demographic factors among all the comparisons. Even in multivariate analysis additionally adjusted for the clinical factors, the significance remained among the comparisons between the second and the third studies (OR 4.45, 95% CI 1.14–17.40) and between the first and the third studies (OR 4.45, 95% CI 1.72–11.50), although it disappeared for the comparison between the first and the second studies (OR 2.54, 95% CI 0.96–6.70) (Table 5).

**Table 4:** The association between WMHL severity at the last study and BG-PVS time-course augmentation, BG-PVS grade at the last study, and demographic and clinical factors

		WMHL at the last study							
		Low grade *n* = 329	High grade *N* = 87	Univariate		Multivariate 1		Multivariate 2	
				Odds ratio	*p* value	Odds ratio	*p* value	Odds ratio	*p* value
BG-PVS augmentation	[Present]	28 (8.5%)	20 (23.0%)	3.21 (1.71–6.04)	< 0.001	2.22 (1.07–4.59)	0.03	2.24 (0.93–5.42)	0.07
BG-PVS at the last study	[High grade]	14 (4.3%)	16 (18.4%)	5.07 (2.37–10.90)	< 0.001	3.31 (1.40–7.83)	0.006	2.61 (0.88–7.70)	0.08
Age-defined group	[Older]	155 (47.1%)	65 (74.7%)	3.32 (1.95–5.63)	< 0.001	3.10 (1.80–5.35)	< 0.001	2.52 (1.29–4.91)	0.007
Sex	[Male]	145 (44.1%)	37 (42.5%)	0.94 (0.58–1.51)	0.80	0.90 (0.54–1.51)	0.69	0.89 (0.48–1.66)	0.72
Current smoker	[Present]	59 (18.0%)	18 (20.9%)	1.20 (0.67–2.17)	0.54				
Hypertension	[Present]	144 (44.0%)	52 (60.5%)	1.94 (1.20–3.15)	0.007			1.06 (0.56–2.00)	0.86
Diabetes mellitus	[Present]	36 (11.0%)	12 (14.0%)	1.31 (0.65–2.64)	0.45				
Plasma sugar (mg/dl)	Mean (SD)	104.7 (20.3)	112.5 (35.7)	1.01(1.00–1.02)	0.01			1.01 (1.00–1.02)	0.04
Office systolic BP (mmHg)	Mean (SD)	138.0 (19.7)	145.3 (21.3)	1.02 (1.01–1.03)	0.004			1.01 (0.99–1.03)	0.24
Office diastolic BP (mmHg)	Mean (SD)	77.1 (12.2)	79.8 (13.2)	1.02 (1.00–1.04)	0.08				
24-h Ambulatory BP (2nd examination)									
Daytime mean (mmHg)	Mean (SD)	103.2 (11. 8)	109.0 (12.6)	1.04 (1.02–1.06)	< 0.001			1.02 (0.99–1.06)	0.28
Nighttime mean (mmHg)	Mean (SD)	91.3 (12.3)	97.5 (10.3)	1.04 (1.02–1.07)	< 0.001			1.01 (0.97–1.04)	0.67
Nighttime BP fall (mmHg)	Mean (SD)	11.4 (8.7)	9.7 (10.2)	0.98 (0.95–1.01)	0.18				

*BG-PVS* perivascular space in the basal ganglia, *BP* blood pressure, *WMHL* white matter hyperintensity lesion

**Table 5:** The association between WMHL severity at the last study and BG-PVS time-course augmentation, BG-PVS grade at the last study, and demographic and clinical factors in the comparisons among the two MRI studies

		WMHL at each the last study							
		Low grade	High grade	Univariate		Multivariate 1		Multivariate 2	
				Odds ratio	*p* value	Odds ratio	*p* value	Odds ratio	*p* value
Comparison between the first and second studies *N* = 350									
BG-PVS augmentation	[Present]	20 (6.9%)	12 (19.4%)	3.22 (1.48–6.99)	0.003	2.73 (1.10–6.73)	0.03	2.54 (0.96–6.70)	0.06
BG-PVS at the second study	[High grade]	14 (4.9%)	10 (16.1%)	3.76 (1.59–8.93)	0.003	2.23 (0.82–6.08)	0.12	2.07 (0.67–6.38)	0.21
Age-defined group	[Older]	127 (44.1%)	46 (74.2%)	3.64 (1.97–6.74)	< 0.001	3.58 (1.91–6.73)	< 0.001	2.90 (1.44–5.83)	0.003
Sex	[Male]	123 (42.7%)	26 (41.9%)	0.97 (0.56–1.69)	0.91	0.91 (0.50–1.67)	0.77	0.87 (0.46–1.65)	0.66
Hypertension	[Present]	130 (45.5%)	37 (60.7%)	1.85 (1.05–3.25)	0.03			1.03 (0.53–2.00)	0.93
Plasma sugar (mg/dl)	Mean (SD)	104.4 (19.8)	110.7 (28.9)	1.01 (1.00–1.02)	0.04			1.01 (1.00–1.02)	0.22
Office systolic BP (mmHg)	Mean (SD)	137.6 (19.7)	147.0 (19.4)	1.02 (1.01–1.04)	0.001			1.02 (1.00–1.03)	0.07
24-h Ambulatory BP (2nd examination)									
Daytime mean (mmHg)	Mean (SD)	103.2 (11.7)	109.5 (12.5)	1.04 (1.02–1.07)	< 0.001			1.02 (0.98–1.05)	0.37
Nighttime mean (mmHg)	Mean (SD)	91.3 (12.2)	98.2 (10.4)	1.05 (1.02–1.07)	< 0.001			1.01 (0.98–1.05)	0.55
Comparison between the second and third studies *N* = 304									
BG-PVS augmentation	[Present]	6 (2.4%)	7 (12.5%)	5.76 (1.86–17.90)	0.002	3.67 (1.08–12.50)	0.04	4.45 (1.14–17.40)	0.03
BG-PVS at the third study	[High grade]	12 (4.8%)	9 (16.1%)	3.77 (1.50–9.44)	0.005	2.51 (0. 90–7.04)	0.08	2.28 (0.72–7.22)	0.16
Age-defined group	[Older]	111 (44.8%)	38 (67.9%)	2.61 (1.41–4.82)	0.002	2.38 (1.27–4.46)	0.007	2.15 (1.04–4.47)	0.04
Sex	[Male]	107 (43.1%)	23 (41.1%)	0.92 (0.51–1.65)	0.78	0.85 (0.45–1.60)	0.62	0.75 (0.38–1.49)	0.41
Hypertension	[Present]	110 (44.7%)	34 (61.8%)	2.00 (1.10–3.64)	0.02			1.14 (0.56–2.31)	0.72
Plasma sugar (mg/dl)	Mean (SD)	104.4 (19.2)	107.8 (22.8)	1.01 (1.00–1.02)	0.25			1.00 (0.99–1.02)	0.58
Office systolic BP (mmHg)	Mean (SD)	138.0 (19.7)	147.5 (20.9)	1.02 (1.01–1.04)	0.002			1.01 (1.00–1.03)	0.16
24-h Ambulatory BP (2nd examination)									
Daytime mean (mmHg)	Mean (SD)	103.1 (11.7)	108.7 (12.2)	1.04 (1.01–1.07)	0.003			1.02 (0.98–1.06)	0.37
Nighttime mean (mmHg)	Mean (SD)	91.2 (12.0)	97.1 (9.8)	1.04 (1.02–1.07)	0.002			1.01 (0.97–1.05)	0.58
Comparison between the first and third studies *N* = 368									
BG-PVS augmentation	[Present]	24 (8.2%)	20 (26.7%)	4.08 (2.11–7.89)	< 0.001	3.09 (1.45–6.57)	0.003	4.45 (1.72–11.50)	0.002
BG-PVS at the third study	[High grade]	13 (4.4%)	14 (18.7%)	4.94 (2.21–11.00)	< 0.001	2.85 (1.14–7.12)	0.02	1.65 (0.49–5.52)	0.42
Age-defined group	[Older]	139 (47.4%)	55 (73.3%)	3.05 (1.74–5.34)	< 0.001	2.79 (1.56–4.98)	< 0.001	2.16 (1.04–4.49)	0.04
Sex	[Male]	132 (45.1%)	31 (41.3%)	0.86 (0.51–1.44)	0.56	0.76 (0.43–1.33)	0.33	0.60 (0.29–1.21)	0.16
Hypertension	[Present]	126 (43.3%)	45 (60.8%)	2.03 (1.21–3.42)	0.008			1.26 (0.62–2.56)	0.52
Plasma sugar (mg/dl)	Mean (SD)	104.8 (19.9)	111.4 (34.0)	1.01 (1.00–1.02)	0.04			1.01 (1.00–1.02)	0.06
Office systolic BP (mmHg)	Mean (SD)	138.4 (19.9)	145.8 (21.9)	1.02 (1.00–1.03)	0.007			1.01 (0.99–1.03)	0.28
24-h ambulatory BP (2nd examination)									
Daytime mean (mmHg)	Mean (SD)	103.0 (11.7)	108.3 (12.6)	1.04 (1.01–1.06)	0.005			1.02 (0.98–1.06)	0.34
Nighttime mean (mmHg)	Mean (SD)	91.1 (12.0)	96.8 (10.0)	1.04 (1.01–1.07)	0.002			1.01 (0.97–1.05)	0.74

*BG-PVS* perivascular space in the basal ganglia, *BP* blood pressure, *WMHL* white matter hyperintensity lesion

## Discussion

This study revealed that both male sex and lower nighttime BP fall were independently associated with the BG-PVS time-course augmentation. As far as we know, this is the first study to evaluate the clinical factors that are associated with BG-PVS time-course augmentation. This study also implied that the time-course augmentation of BG-PVS visualization would be a meaningful image finding that was related to high-grade WMHL in the subsequent MRI.

This study showed that lower nighttime BP fall and male sex were significant clinical factors related to BG-PVS time-course augmentation. Other well-known cardiovascular risk factors including medical history and blood test abnormality did not have a significant association with BG-PVS time-course augmentation, which was consistent with a previous study [29]. The association between ambulatory blood pressure and EPVS at a point in time has been reported and EPVS is presumed to reflect damage of endothelial cells in small vessels [30, 31]. However, the association between BG-PVS time-course change and ambulatory blood pressure has not been reported previously. Ambulatory blood pressure is a more valuable predictor for cardiovascular events than office BP [32]. In a normal circadian rhythm, nighttime BP is lower than daytime BP; that is, an approximately 10%–20% BP fall is observed during sleep [33]. An insufficient nighttime BP fall is related to the subsequent cardiovascular event [34]. Although the mechanism of this association remains unproved, worsening of arterial stiffness caused by nighttime-lasting high blood pressure was proposed as the mechanism [35]. For CSVD, the pathology includes hyaline or hyperplastic arteriolosclerosis caused by hypertension [7]. It has also been reported that the low nighttime BP fall rate was related to CSVD [36]. Riba-Llena et al*.* reported that PVS enlargement was also related to arterial stiffness [37]. They discussed the mechanism as follows: increased arterial stiffness in large arteries causes high pulsatility in peripheral small arteries, which facilitates endothelial cell injury leading to interstitial fluid leakage and PVS enlarges by failing to drain the redundant interstitial fluid [37]. Although the causal relation cannot be proven in this study because ABPM was performed at the intermediate point in the study period, the association between BG-PVS time-course augmentation and lower nighttime BP fall might reflect underlying worsening arterial stiffness, endothelial cell injury in small vessels and redundant interstitial fluid leakage. Further studies are needed to reveal the causal relation between BG-PVS time-course augmentation and the abnormality in 24-h ambulatory blood pressure, and the mechanism behind this relationship. In addition, male sex was associated with BG-PVS time-course augmentation even when adjusted for arteriosclerosis factors. Among previous studies in which the association between sex and BG-PVS severity at a single point in time was examined, one study revealed that severe BG-PVS was more frequent in men [38], and other studies revealed no significance in the association [10, 39]. That is, the sex difference effect has remained uncertain. Moreover, the association between sex and BG-PVS time-course augmentation has not been adequately examined, so further studies are also needed to verify any difference due to sex.

This study also revealed that BG-PVS time-course augmentation was an image finding significantly associated with subsequent severe WMHL, when adjusted for BG-PVS severity at the same point in time, sex, and age. Although previous population-based studies have already reported that BG-PVS and WMHL time-course augmentation were associated with each other and that their severity at the baseline promoted subsequent WMHL time-course augmentation [29, 40, 41], the relation between the BG-PVS time-course augmentation and the subsequent WMHL severity has not been reported previously. EPVS and WMHL at a single point in time are recognized as image findings for CSVD [8]. EPVS in basal ganglia is associated with arteriolosclerosis [42], and the degree of its visualization is also associated with WMHL [9–12, 43]. Assuming that WMHL reflects the severity of CSVD, the results of this study suggest that BG-PVS time-course augmentation would be a meaningful image finding for CSVD with a hypertensive vasculopathy background. When the clinical factors were adjusted, the significance of BG-PVS time-course augmentation as well as BG-PVS severity in the last study disappeared, which indicated that these image findings were influenced by clinical factors, particularly a history of hypertension. However, BG-PVS time-course change remained as a relatively large odds ratio, and the difference in odds ratio between the univariate and multivariate analysis was smaller than that of BG-PVS severity in the last study. Additionally, in some of the accessory analyses, BG-PVS time-course augmentation remained significant even when clinical factors were adjusted, although the significance of the BG-PVS severity at a single point in time disappeared. This indicated that BG-PVS time-course augmentation was less influenced by clinical factors than BG-PVS severity at a single point in time, and that it was probable that it would be a notable image finding for CSVD. From the results of this study, BG-PVS time-course augmentation would reflect peripheral small vessel impairment and be a meaningful finding suggesting the progression of CSVD.

This study has some limitations. First, not all participants had three MRI studies, and the participants with only one MRI study were excluded from the study population. In addition, even among the study population, not all participants recorded adequate clinical information in each analysis. Moreover, because ABPM was performed only once, we can’t exclude the possibility that it is not enough to serve as a representative value in this study period. Second, we examined image findings acquired by the 0.3 T MRI system. It is possible that small PVS could not be recognized, as these would be described by a higher magnetic field system. Third, we employed a semi-quantitative visual scale for BG-PVS visualization and WMHL severity and we also assessed BG-PVS time-course augmentation by eye; this route was less objective than widely used computer-based automatic methods. However, all the raters were blinded to any clinical information when assessing image findings. For inter-tater reliability assessment, the kappa coefficients were slightly low, although observed agreement rates were high; 0.93 for BG-PVS severity, 0.90 for WMHL severity, and 0.88 for BG-PVS augmentation. We think that this relatively low kappa coefficients were influenced by the low prevalence of severe BG-PVS, severe WMHL and BG-PVS augmentation in this study population. Byrt T, et al. reported that Cohen’s kappa coefficient decreases as the prevalence index increases [44]. For the indices they proposed, the prevalence index, the bias index, and prevalence-adjusted bias-adjusted kappa were  – 0.725, 0.025 and 0.85 for BG-PVS severity,  – 0.55, 0.10, and 0.80 for WMHL severity, and  – 0.625, 0.075 and 0.75 for BG-PVS augmentation. So, we think the reliability of the image assessment is ensued. This study validated the reliability of these visual semi-quantitative or qualitative scales, which is acceptable in daily medical practice. Finally, we did not evaluate microbleeds, lacune, and recent small subcortical infarcts, which are image findings indicating CSVD [8]. Further studies with larger study populations using computer-based, more objective methods for high-resolution magnetic resonance (MR) images are vitally necessary to verify the results of this study.

In conclusion, this study revealed that a lower nighttime BP fall was a significant clinical factor related to BG-PVS augmentation. Additionally, the BG-PVS time-course augmentation was associated with subsequent WMHL severity, which implied that the time-course BG-PVS change would be a meaningful image finding for CSVD. This suggests that we should note the time-course change in addition to the severity at a single point in time for BG-PVS when interpreting brain images in an elderly population.

## Abbreviations

ABPM: Ambulatory blood pressure monitoring
BG-PVS: Perivascular space in the basal ganglia
CI: Confidence interval
CSVD: Cerebral small vessel disease
FLAIR: Fluid-attenuated inversion recovery
OR: Odds ratio
PVS: Perivascular space
T1WI: T1-weighted image
T2WI: T2-weighted image
WMHL: White matter hyperintensity lesion
